# The History of a Case in Which Animals Were Found in Blood Drawn from the Veins of a Boy; with Remarks

**Published:** 1834-01-01

**Authors:** 


*The History of a Case in which Animals were found in Blood drawn from the Veins of a Boy; with Remarks.* By J. Stevenson Bushnan, f.l.s., Surgeon to the Dumfries Dispensary.—London, 1833. 8vo. pp. 74.

On the 4th of June, 1833, Mr. Bushnan bled a boy suffering under influenza, who was said to have worms in his blood. "I took," says our author, "about six ounces of blood from his left arm, in which I could not at first discover anything preternatural, but which I carefully covered with a bason, placing it in such a manner that it could not be disturbed without my knowledge. On returning, one hour afterwards, I found five animals swimming in the serum of the blood, all most vigorous and lively." (P. 6.) Mr. Rhind, of Edinburgh, to whom some of these worms were sent, says that they exactly correspond in structure and colour, and size, to the larvæ of the *Tipula oleracea*, which, in summer, are so abundantly found in ditch and river water." (P. 8.)

Our readers will easily see that there is a possibility of the worms having been supposititious. Mr. Bushnan, however, thinks this highly improbable, and proceeds to give similar cases from medical authors; which are followed by some ingenious conjectures as to the origin of the proper entozoa of the human body. Our author is of opinion, with Rudolphi and Bremser, "that the entozoa in general are generated primarily, not from ova at all, but spontaneously in each organ in which they are found." (P. 44.) He observes that this is not exactly the doctrine of equivocal generation, by which is meant the production of life in matter previously existing. According to the theory of Rudolphi and Bremser, the living matter is secreted by the various organs of the human body, that is, smaller living beings are formed by the separation of certain living particles from larger ones. The conjecture which appears to us the most natural is, that the ova of the parasitic animals are received into the body with the air or food. On this point Mr. Bushnan says,

"One of the last and most ingenious attempts to trace a real parasite of the human body to the ingesta, was made by Dr. Chisholm, with respect to the Filaria Medinensis, or skin-worm; and in this view of the matter he is borne out, not only by the authority of Bremser, Bruce, Chardin, Dampier, Dubois, and numerous other writers, but by some very forcible arguments derived from his own observation.* If, however, this fact were quite established, it would follow, not that such was the case with the entozoa in general, but only that the worm in question is not really one of these, but belongs, in fact, to the second class of animals, with which, as I have already shown, the various organs of the human body are so frequently infested." (P. 47.)

To us it appears that Dr. Chisholm's investigations are a severe blow to the Rudolphian theory, and that a few more Chisholms would knock it on the head altogether; for an exception is hard to be borne by a rule built on conjecture, and contrary to analogy.

Mr. Bushnan supposes that, in the case of his patient, the worms sprung from ova introduced from without, and there-

* "Chisholm; Ed. Med. and Surg. Journal, 1815."

fore are not to be numbered among the genuine parasites of the human body.

This little work is creditable to the talents of Mr. Bushnan; but would have been better calculated for an article in a journal than a substantive book.

